# Postoperative Shock After Bariatric Surgery: The Role of Point-of-Care Cardiac Ultrasound

**DOI:** 10.7759/cureus.87179

**Published:** 2025-07-02

**Authors:** Lucas Ricchetti, Thiago Viçoso, Jorge Hamilton Garcia, Chiara Tessmer

**Affiliations:** 1 Cardiac Anesthesia Department, Hospital SOS Cardio de Santa Catarina, Florianópolis, BRA

**Keywords:** bariatric surgery complications, perioperative cardiac evaluation, point-of-care cardiac ultrasound, postoperative cardiac dysfunction, takotsubo cardiomyopathy

## Abstract

On the fifth day after surgery, during the postoperative period of bypass gastric surgery, a patient returned to the emergency department complaining of acute chest pain. Initially, acute coronary injury was suspected. The main hypothesis was ruled out after angiography revealed no thrombotic evidence. Thus, a diagnosis of Takotsubo syndrome was considered after the on-call anesthesiology team performed a bedside echocardiogram in the hemodynamics room. This report highlights the importance of cardiac ultrasound as a tool to detect anomalies and guide treatment and prognosis.

## Introduction

Takotsubo syndrome, or Takotsubo cardiomyopathy, is characterized by transient systolic and diastolic left ventricular dysfunction with various wall motion abnormalities [1]. It predominantly affects postmenopausal women and is often preceded by physical or emotional stressors [1]. Although Takotsubo syndrome presents with symptoms similar to acute myocardial infarction, such as chest pain and shortness of breath, it occurs without coronary artery obstruction [2]. In the acute phase, clinical presentation, electrocardiographic findings, and biomarker profiles are often similar to those of acute coronary syndrome (ACS) [3]. Although most patients fully recover within weeks or months, some may experience severe heart failure and even death [4]. Early diagnosis and appropriate treatment are essential to ensure full recovery and prevent complications.

## Case presentation

A 30-year-old female patient presented on the fifth postoperative day following gastric bypass surgery (BMI 35 kg/m^2^), with no significant medical history, presented to the emergency department with sudden retrosternal chest pain, described as a compressive sensation, shortness of breath, which had started one hour prior to hospital arrival. On physical examination, she appeared anxious and uncomfortable, with a blood pressure of 140/90 mmHg and a heart rate of 110 bpm. Cardiovascular examination revealed a soft murmur and bibasilar crackles, and her oxygen saturation was 90% on room air.

**Figure 1:**
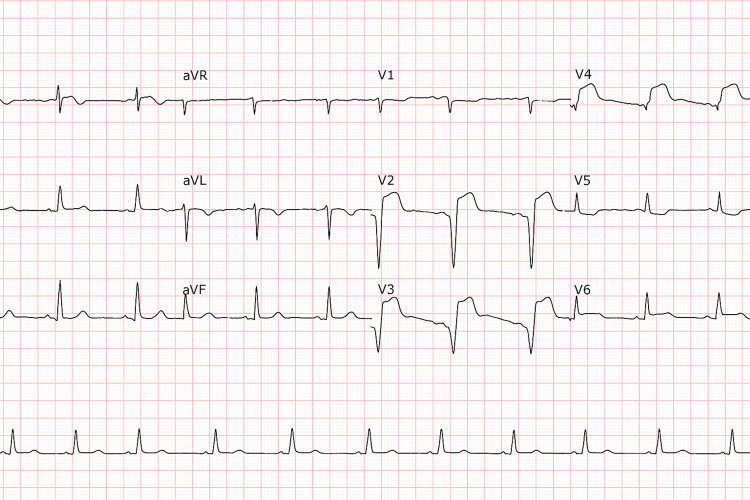
Electrocardiogram performed upon admission Sinus rhythm and heart rate of 68 bpm, with ST-segment elevation in leads V1-V4, were suggestive of anterior ischemia. There were no Q waves or conduction abnormalities.

She was immediately placed on the chest pain protocol, with ECG and troponin measurements performed. The laboratory tests showed no evidence of electrolyte abnormalities upon admission. At the time, she was tolerating a liquid diet without difficulty, and there were no clinical or laboratory signs of malabsorption. The ECG showed ST-segment elevation in the anterior leads, and troponin levels were three times the upper limit of normal, suggesting acute myocardial injury (Figure 1). Based on clinical and laboratory findings, ACS was initially suspected.

The patient was urgently transferred to the coronary angiography suite, and anesthesiology was consulted. Thrombolysis was not indicated in this case because the hospital is equipped with an interventional cardiac catheterization laboratory, and primary percutaneous coronary intervention (PCI) could be performed within the recommended 90-minute window. The angiogram revealed normal coronary arteries with no significant obstructions. However, despite light sedation with midazolam 2 mg and fentanyl 50 mcg, the patient developed hypotension, tachypnea, and bibasilar crackles, suggestive of acute pulmonary edema.

Based on clinical findings, the anesthesiology team decided to perform a bedside echocardiogram, which revealed apical akinesia and basal hyperkinesia of the left ventricle with an ejection fraction of 20%, according to Simpson’s method (Video 1). No evidence of left ventricular outflow tract obstruction (LVOTO) was noted on initial evaluation. A left ventriculography was requested, which showed ballooning of the left ventricle (Video 2). Based on the imaging findings, the diagnosis of Takotsubo syndrome was established. During the acute phase, including diagnosis and hemodynamic stabilization, the patient was managed exclusively by the anesthesiology team.

**Video 1:** Apical ventricular akinesia

**Video 2:** Ventriculography showing apical ballooning of the left ventricle

After the diagnosis of Takotsubo syndrome, the patient was transferred to the coronary care unit with dobutamine, norepinephrine, and bilevel positive airway pressure (BiPAP) support. The patient did not have a central venous line and was managed with a peripheral intravenous line. Continuous invasive arterial pressure monitoring was performed via a radial arterial catheter. At presentation, her initial mean arterial pressure was approximately 50 mmHg. After initiation of pharmacologic support, it stabilized at around 80 mmHg. She received low doses of vasoactive agents: norepinephrine at 0.2 mcg/kg/min and dobutamine at 3.5 mcg/kg/min. Intravenous fluid resuscitation was administered judiciously to avoid volume overload in the context of potential left ventricular outflow tract obstruction. During hospitalization, the patient experienced rapid clinical deterioration, progressing to cardiogenic shock and acute respiratory failure, which required endotracheal intubation and mechanical ventilation. In response to hemodynamic instability, vasopressor support with norepinephrine was initiated. Following the diagnosis of left ventricular outflow tract (LVOT) obstruction on echocardiography, inotropic agents were avoided, as they could exacerbate the dynamic obstruction.

Despite the need for intensive hemodynamic support with norepinephrine and dobutamine due to cardiogenic shock, the patient remained hemodynamically stable after initial stabilization and did not experience any arrhythmias, thromboembolic events, or further organ dysfunction. Serial laboratory tests and echocardiography showed progressive improvement in cardiac function. She was successfully weaned off vasopressors, extubated without complications, and transferred to the general ward in stable condition. Cardiac function gradually improved, allowing withdrawal of vasoactive drugs and ventilatory support. She was discharged a few days later with cardiology follow-up and stress management recommendations.

## Discussion

The patient presented with typical chest pain after an acute emotional stress likely triggered by her bariatric surgery, initially raising suspicion for ACS, on her fifth postoperative day. ECG and elevated troponins supported this hypothesis. However, the absence of coronary obstruction on angiography ruled out a traditional ACS. The etiopathogenic mechanisms underlying Takotsubo syndrome remain poorly understood. However, a transient cardiac dysfunction appears to be triggered by a surge in catecholamines in response to emotional or physical stressors. Early measurements have demonstrated catecholamine levels comparable to, or even exceeding, those observed in acute myocardial infarction, reaching up to 34 times baseline values [5].

The natural history, management, and outcome of Takotsubo cardiomyopathy are not fully understood [3]. Coronary microcirculation is innervated by brainstem neurons that mediate vasoconstriction, supporting the theory of neurogenic myocardial stunning due to microvascular dysfunction in Takotsubo syndrome [3].

Our case reinforces that troponin levels and ECG changes alone are insufficient to differentiate Takotsubo syndrome from ACS, as over 80% of Takotsubo patients have elevated troponins. Early coronary angiography remains necessary to exclude obstructive coronary artery disease [3].

Diagnosis relies on imaging findings on left ventricular angiogram or echocardiography, including apical and mid-ventricular akinesia, hypokinesia, or dyskinesia, with or without basal hyperkinesia, giving the characteristic "apical ballooning" appearance and causing dynamic obstruction of the LVOT [2]. Such alterations can be identified through non-invasive examinations, such as echocardiography, cardiac magnetic resonance imaging, and myocardial scintigraphy, as well as through invasive tests, including coronary angiography and ventriculography [3,6].

Echocardiography is usually performed prior to coronary angiography in symptomatic patients without ST-segment elevation, and in cases with ST-segment elevation when the risks of angiography and angioplasty outweigh the potential benefits. Typically, the area of left ventricular dysfunction extends beyond the territory supplied by a single epicardial coronary artery, and this pattern of wall motion abnormality may suggest a diagnosis of Takotsubo cardiomyopathy [7]. In this case, the patient presented with ST-segment elevation and symptoms suggestive of myocardial ischemia; therefore, the next step in medical management was percutaneous coronary intervention within the first 90 minutes from first medical contact, in a hemodynamically equipped facility capable of performing coronary reperfusion therapy [8].

The anesthesiologist plays a pivotal role in the use of point-of-care ultrasound (POCUS), particularly in intraoperative and perioperative settings. As frontline providers during surgery and in critical care environments, anesthesiologists are uniquely positioned to perform bedside cardiac ultrasound promptly in response to hemodynamic instability. In this case, the anesthesiology team conducted a focused cardiac ultrasound during the angiography procedure, which revealed characteristic findings of apical akinesia and basal hyperkinesia-hallmarks of Takotsubo cardiomyopathy. The rapid deployment of POCUS was essential in ruling out acute coronary syndrome and guiding immediate management strategies. This emphasizes how the integration of POCUS into anesthesiology practice enhances diagnostic accuracy, supports timely therapeutic decisions, and ultimately improves patient outcomes in acute perioperative scenarios [9].

Currently, there is no standardized treatment for Takotsubo syndrome. Immediate cardiac catheterization is crucial, as diagnosis requires ruling out obstructive coronary artery disease [3]. Treatment involves hemodynamic support with intravenous fluid, vasopressors, and the management of complications such as cardiogenic shock, intracavitary thrombi, heart failure, and arrhythmias. Most patients have an excellent prognosis with full ventricular recovery occurring within six to eight weeks. In-hospital mortality is below 2%. Repeat echocardiography is essential to confirm the complete recovery of left ventricular function and to detect potential complications associated with Takotsubo cardiomyopathy [10].

In this case, dual antiplatelet therapy was initiated under the initial acute coronary syndrome hypothesis. Although the patient improved, she required intensive support due to cardiogenic shock. She progressed without complications despite requiring intensive hemodynamic support during hospitalization.

## Conclusions

This case highlights the importance of considering Takotsubo syndrome in the differential diagnosis of postoperative chest pain, especially when clinical and laboratory findings initially suggest acute coronary syndrome. The absence of significant coronary obstruction and the typical appearance of apical ballooning on the left ventriculogram, together with the prompt use of POCUS by the anesthesiology team, were crucial in establishing the correct diagnosis. The anesthesiologist, as a perioperative specialist, is uniquely positioned to perform POCUS in situations of hemodynamic instability, enabling rapid assessment and guiding immediate clinical decisions. This reinforces the relevance of incorporating POCUS into anesthetic practice to enhance diagnostic accuracy and optimize patient outcomes in acute perioperative scenarios.
